# Insulin-like growth factor-independent insulin-like growth factor binding protein 3 promotes cell migration and lymph node metastasis of oral squamous cell carcinoma cells by requirement of integrin β1

**DOI:** 10.18632/oncotarget.5995

**Published:** 2015-10-19

**Authors:** Yi-Chen Yen, Jenn-Ren Hsiao, Shih Sheng Jiang, Jeffrey S. Chang, Ssu-Han Wang, Ying-Ying Shen, Chung-Hsing Chen, I-Shou Chang, Jang-Yang Chang, Ya-Wen Chen

**Affiliations:** ^1^ National Institute of Cancer Research, National Health Research Institutes, Miaoli, Taiwan; ^2^ Department of Otolaryngology, National Cheng Kung University Hospital, College of Medicine, National Cheng Kung University, Tainan, Taiwan; ^3^ National Institute of Cancer Research, National Health Research Institutes, Tainan, Taiwan; ^4^ Pathology Core Laboratory, National Health Research Institutes, Miaoli, Taiwan; ^5^ Institute of Population Health Sciences, National Health Research Institutes, Miaoli, Taiwan; ^6^ Department of Internal Medicine, National Cheng Kung University Hospital, College of Medicine, Tainan, Taiwan; ^7^ Graduate Institute of Basic Medical Science, China Medical University, Taichung, Taiwan

**Keywords:** oral squamous cell carcinoma, lymph node metastasis, migration, insulin-like growth factor binding protein 3, integrin β1

## Abstract

Frequent metastasis to the cervical lymph nodes leads to poor survival of patients with oral squamous cell carcinoma (OSCC). To understand the underlying mechanisms of lymph node metastasis, two sublines were successfully isolated from cervical lymph nodes of nude mice through *in vivo* selection, and identified as originating from poorly metastatic parental cells. These two sublines specifically metastasized to cervical lymph nodes in 83% of mice, whereas OEC-M1 cells did not metastasize after injection into the oral cavity. After gene expression analysis, we identified insulin-like growth factor binding protein 3 (IGFBP3) as one of the significantly up-regulated genes in the sublines in comparison with their parental cells. Consistently, meta-analysis of the public microarray datasets and IGFBP3 immunohistochemical analysis revealed increased both levels of IGFBP3 mRNA and protein in human OSCC tissues when compared to normal oral or adjacent nontumorous tissues. Interestingly, the up-regulated IGFBP3 mRNA expression was significantly associated with OSCC patients with lymph node metastasis. IGFBP3 knockdown in the sublines impaired and ectopic IGFBP3 expression in the parental cells promoted migration, transendothelial migration and lymph node metastasis of orthotopic transplantation. Additionally, ectopic expression of IGFBP3 with an IGF-binding defect sustained the IGFBP3-enhanced biological functions. Results indicated that IGFBP3 regulates metastasis-related functions of OSCC cells through an IGF-independent mechanism. Furthermore, exogenous IGFBP3 was sufficient to induce cell motility and extracellular signal-regulated kinase (ERK) activation. The silencing of integrin β1 was able to impair exogenous IGFBP3-mediated migration and ERK phosphorylation, suggesting a critical role of integrin β1 in IGFBP3-enchanced functions.

## INTRODUCTION

Oral squamous cell carcinoma (OSCC) is one of the most frequent cancers worldwide, with more than half a million patients being diagnosed annually [1]. Patients with lymph node metastasis have a markedly worse prognosis than patients without metastasis. The 5-year overall survival rates for OSCC patients are approximately 80% for patients without lymph node metastasis and 45% for patients with lymph node metastasis [2]. An accurate assessment of the cervical lymph node metastasis status in OSCC not only helps predict the prognosis of patients, but also benefits for the appropriate treatment. Thus, understanding the pathophysiology of lymph node metastasis of OSCC is important for early diagnosis and treatment. However, precise molecular mechanisms of lymph node metastasis have not been elucidated, partly due to the lack of consistent and reproducible animal models.

Cancer metastasis is thought to originate from a small proportion of the cancerous cells in primary tumors. Therefore, screening for a subpopulation of cells with high metastatic potential from a parental tumor cell line in experimental models is a well-defined method for discovering genes that play roles in metastasis, especially that which preferentially occurs in specific organs. Using an orthotopic implantation model of OSCC that spontaneously metastases to the cervical lymph nodes, we have successfully established two independent OSCC sublines. Microarray analysis provided a technical way to investigate the molecular pathways involved in the growth and metastasis of OSCC *in vivo and in vitro* [3]. By analyzing the differentiated gene expression, we identified insulin-like growth factor binding protein 3 (IGFBP3) as one such up-regulated gene that might participate in tumorigenesis and lymph node metastasis of OSCC.

Insulin-like growth factor binding protein 3 is a member of a secretary glycoprotein family that can bind insulin-like growth factor 1 or 2 (IGF1 or IGF2) in circulation and regulate the mitogenic activity of insulin-like growth factor I receptor (IGF1R) [4]. Abnormal expression or malfunction of IGFBP3 is associated with tumor development and progression. Reduced IGFBP3 expression has been reported in several cancers such as lung cancer, hepatocellular carcinoma, ovarian cancer and prostate cancer [5–9]. However, increased IGFBP3 has been demonstrated in some other cancers, including renal cell carcinoma, esophageal carcinoma, breast, colon, pancreatic and cervical cancers [10–15]. Being a suppressor, several studies have confirmed that IGFBP3 suppresses cell adhesion [16], invasiveness of endometrial cancer [17], metastasis in prostate cancer [18], and angiogenesis in head and neck squamous cell carcinoma [19]. In contrast, IGFBP3 has an activity of antioxidation, suppressing reactive oxygen species [20] and promoting epithelial-to-mesenchymal transition and motility [21] for tumor progression. Thus, IGFBP3 may have context-dependent tumor-promoting activities.

Apart from the ability to inhibit or enhance IGF actions, IGFBP3 also exhibits very clear, distinct biological effects independent of the IGF/IGF1R axis. Focusing on IGFBP3-medaited biological effects by cell surface association of IGFBP3 with receptor, IGFBP3 has been proposed as a functional ligand for the serine/threonine kinase type V transforming growth factor-β receptor (TGF-βRV) and interaction of IGFBP3 with TGF-βRV causes cell growth inhibition [22]. Additionally, a putative unconventional death receptor, termed IGFBP-3R was hypothesized to be a death receptor due to its cytoplasmic tail binding to caspase-8 [23]. In contrast, Martin et al. showed that IGFBP3 stimulates growth via increased epidermal growth factor receptor (EGFR) phosphorylation and activation of p44/42 and p38 mitogen activated protein kinase (MAPK)/extracellular signal-regulated kinase (ERK) signaling pathways in breast epithelial cells [24].

Given the diverse connection between IGFBP3 and cancer phenotypes, the functional roles of IGFBP3 in tumorigenesis and lymph node metastasis of OSCC remain vague. So far, only one study reported the positive correlations between the IGFBP3 protein-positive grade in OSCC tissue and the tumor size as well as lymph node metastasis [25]. In this study, by *in vivo* selection of more invasive cells from orthotopic mice model, human cancer tissues and cell based *in vitro* analyses, we have established the functional correlations between IGF-independent IGBBP3 and lymph node metastasis of OSCC.

## RESULTS

### *In vitro* characterization of OSCC sublines established by *in vivo* selection

**Figure 1:**
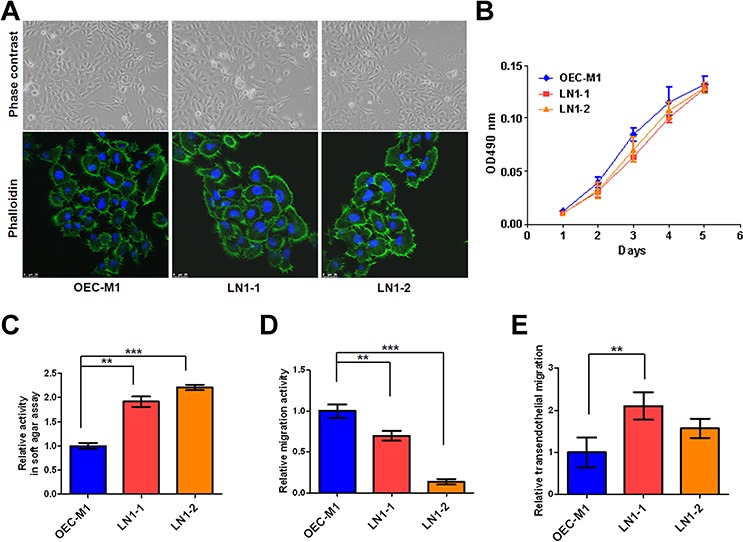
The LN1–1 and LN1–2 sublines demonstrate higher transendothelial migration activity **A.** Cell morphology and cytoskeleton F-actin of OEC-M1 (left panel), LN1–1 (middle panel) and LN1–2 (right panel) cells were observed under a phase contrast microscope at 200 × magnification (upper panel) and viewed under fluorescent microscope at 630 × magnification by staining with Alex Fluro 488 phalloidin (lower panel), respectively. **B.** Representative data shows cell growth in OEC-M1, LN1–1 and LN1–2 cells by MTS assay. **C.** Representative data shows anchorage-independent growth activity for OEC-M1 cells and sublines. The relative activity was determined by normalizing the mean of colonies/per plate in sublines to that in OEC-M1 cells. **D.** Representative data shows the relative migration and **E.** transmigration activities of OEC-M1, LN1–1 and LN1–2 cells. The relative migration/transendothelial migration activity was defined by normalizing the mean of migrated cell/per field in sublines to that in OEC-M1 cells. Bar, SE; **p < 0.01; ***p < 0.001.

The lymph nodes from animals with orthotopic implantation of OEC-M1 cells, a poorly metastastic OSCC cells, were minced and cultured *in vitro* to yield a continuously growing cell mixture. Two sublines, denoted as LN1–1 and LN1–2 cells, were isolated from the cervical lymph nodes of different animals sacrificed on day 42 and 56, respectively. Detection of short tandem repeat (STR) markers was performed and it was found that LN1–1 and LN1–2 cells were derived from their parental OEC-M1 cells (Table S1). The three cell lines grew with typical cobblestone-like epithelialoid morphology and showed no gross difference on plastic surface, when examined under either light microscope or fluorescent confocal microscope with phalloidin staining (Figure 1A). Although the three cells showed similar kinetics of adhesive growth as analyzed by MTS assay (Figure 1B), those two sublines exhibited higher potential of anchorage independent growth than OEC-M1 cells in soft agar assay (Figure 1C). To test the migratory properties, we performed the transwell assay and unexpected found that the migration activities in sublines were significantly decreased when compared to their parental cells (Figure 1D). Furthermore, we conducted transendothelial migration assay to investigate the ability of cancer cells to pass across the lymphatic endothelium. LN1–1 sublines showed markedly higher capability for transendothelial migration when compared to OEC-M1 cells (Figure 1E).

### Enhanced tumor growth in OSCC sublines

**Figure 2:**
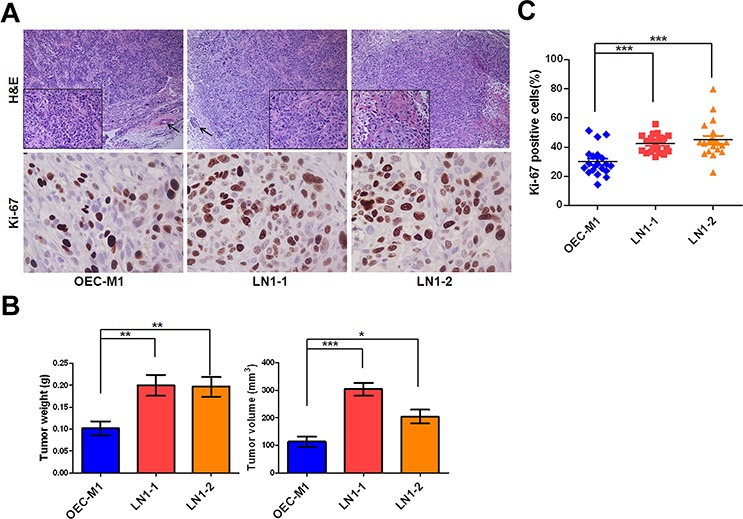
LN1–1 and LN1–2 sublines show increased *in vivo* tumor growth **A.** The representative fields of histology and Ki-67 expression in OEC-M1 (left panel), LN1–1 (middle panel) and LN1–2 (right panel) tumors by Hematoxylin and Eosin (H&E) staining and immunohistochemistry (IHC) were observed under light field microscope with 100 and 400 × magnifications, respectively. The histological examination from OEC-M1 and LN1–1 tumors shows poorly differentiated cells (lower box with 400 × magnification) and lymphovascular invasion (shown in arrow). The histological examination from LN1–2 tumors shows moderated differentiated squamous cell carcinoma with focal keratinization (lower box). **B.** Quantification of tumor weight (left panel) and volume (right panel) is shown for mice (*n* = 6–7) orthotopically injected with OEC-M1, LN1–1 and LN1–2 cells. **C.** The percentage of positive Ki-67 signals was determined in OEC-M1, LN1–1 and LN1–2 tumors. Bar, SE; *p < 0.05; **p < 0.01; ***p < 0.001.

All of the three OSCC cell lines were highly tumorigenic and developed tumors successfully in all mice (Table 1). Histological examination of those orthotopic tumors demonstrated characteristics of epithelial differentiation (Figure 2A). However, the weight and size of tumors formed varied among three cell lines. The orthotopic tumor weight of mice receiving LN1–1 (*n* = 7) and LN1–2 (*n* = 6) cells were 0.20 ± 0.024g and 0.197 ± 0.022g respectively, both larger than that of their parental OEC-M1 cells (0.107 ± 0.016g, *n* = 6) (Figure 2B). Similarly, we observed larger tumor volumes in mice bearing sublines than in those bearing the parental cells (Figure 2B). Consistent to these findings, more Ki-67 positive cells cells were demonstrated in LN1–1 (42.3 ± 1.38%) and LN1–2 (45 ± 2.77%) orthotopic tumors relative to the OEC-M1 tumors (29.94 ± 2.12%) (Figures 2A and 2C). Our data suggest that the *in vivo* selected OSCC sublines have elevated tumor growth when compared to the parental cells.

**Table 1:** Tumor formation and spontaneous lymph node metastases in mice injected with oral squamous cell carcinoma (OSCC) cells

Groups	Tumors/No of mice	Tumorigenesis (%)	Metastasis/No of mice	Metastasis (%)
OEC-M1	6/6	100	0/6	0
LN1–1	7/7	100	5/6	83.3
LN1–2	6/6	100	5/6	83.3
LN1–1 GFP	10/10	100	7/10	70
LN1–1 IGFBP3 sh4	9/9	100	3/9	30
LN1–1 IGFBP3 sh5	10/10	100	4/10	40
OEC-M1 PB	8/8	100	1/8	12.5
OEC-M1 IGFBP3	8/8	100	3/8	37.5
OEC-M1 IGFBP3 GGG	7/7	100	3/7	42.9

### Higher potential of lymph node metastasis in OSCC sublines

**Figure 3:**
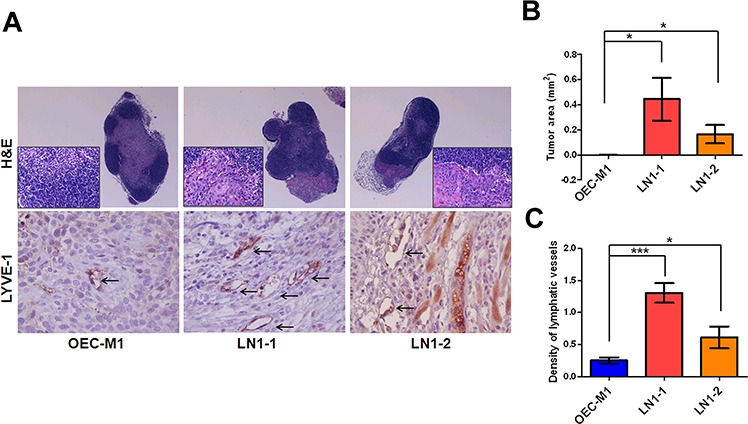
Higher lymphangiogenesis in orthotopic tumors generated from the OSCC sublines **A.** H&E staining of lymph nodes from mice with orthotopic tumors generated from OEC-M1 (left panel), LN1–1 (middle panel) and LN1–2 (right panel) cells, was observed under microscope at 40 and 400 × magnification. The intratumoral lymphatic vessels in orthotopic OEC-M1 (left panel), LN1–1 (middle panel) and LN1–2 (right panel) tumors were stained using anti-LYVE-1 and observed with 400 × magnification (lower panel). The arrow indicates lymphatic vessels. **B.** Quantification of tumor area in lymph nodes of mice with orthotopic growth of OEC-M1, LN1–1 and LN1–2 cells. The data were expressed as mean tumor area of lymph nodes in mice (*n* = 4–6) bearing orthotopic tumors. **C.** Quantification of tumor lymphatic vessels by IHC with anti-LYVE1. The data were expressed as mean density of LYVE-1 positive number per microscopic field. Bar, SE; *p < 0.05; ***p < 0.001.

By Hematoxylin and Eosin (H&E) staining, we noted that most metastatic tumor cells were found from the marginal sinus to the paracortex sinus of the lymph nodes (Figure 3A). Importantly, 83% of the animals (*n* = 6) had lymphatic metastasis after orthotopic injection of LN1–1 or LN1–2 cells, while no animals with lymph node metastasis after injection of OEC-M1 cells were observed (Table 1). The average tumor area of lymph nodes was larger in mice injected with LN1–1 and LN1–2 cells (Figure 3B). To explore whether the higher incidence of lymphatic metastasis was accompanied by more intratumoral lymphatics, quantification of lymphatic vessel endothelial hyaluronic acid receptor 1 (LYVE-1) positive areas demonstrated that intratumoral lymphatics were more abundant in tumors of mice injected with LN1–1 (1.31 ± 0.15 vessels/per filed) and LN1–2 cells (0.61 ± 0.17 vessels/per field) than that in OEC-M1 cells (0.25 ± 0.051 vessels/per field) (Figure 3A and 3C). Our data suggest that LN1–1 and LN1–2 cells have higher capability to metastasize to lymph nodes in comparison with OEC-M1 cells.

### Up-regulation of IGFBP3 mRNA and protein in OSCC tissues

To identify the genes related to tumorigenesis and lymphatic metastasis, we performed microarray analysis to obtain differential gene expression between those sublines and parental cells. The dataset is available in GEO/GSE62326. A total of 554 common probes representing 521 genes showed an at least 1.5-fold change of expression in two sublines when compared to the parental one, with 278 being up-regulated and 243 down-regulated (Figure S1). To identify genes with clinical relevance, we further compared our data with another dataset, which was derived from 40 pairs of OSCC tumor with matched nontumourous tissues [26]. Via such a comparison, 101 up-regulated and 8 down-regulated genes were found commonly between two datasets (Figure S1). The top-10 most up-regulated and top-5 most down-regulated genes are as listed in Table 2.

**Table 2:** List of the top 10 up-regulated and top 5 down-regulated genes

De-regulated gene	Description	Fold change
IFI27	Interferon, alpha-inducible protein 27	162
OAS2	ARID1B AT rich interactive domain 1B	21
DHX58	DEXH (Asp-Glu-X-His) box polypeptide 58	10
CFB	Complement factor B	10
ANKRD38	KN motif and ankyrin repeat domains 4	7.2
PDZK1	PDZ domain containing 1	6.6
OAS1	ARID1B AT rich interactive domain 1A	6.5
BST2	Bone marrow stromal cell antigen 2	6.4
IGFBP3	Insulin-like growth factor binding protein 3	5.5
IFI6	Interferon, alpha-inducible protein 6	5.1
PKIA	Protein kinase (cAMP-dependent, catalytic) inhibitor alpha	0.38
SLC47A1	Solute carrier family 47 (multidrug and toxin extrusion), member 1	0.41
CGNL1	Cingulin-like 1	0.45
EPHX1	Epoxide hydrolase 1, microsomal (xenobiotic)	0.48
PGD	Phosphogluconate dehydrogenase	0.53

**Figure 4:**
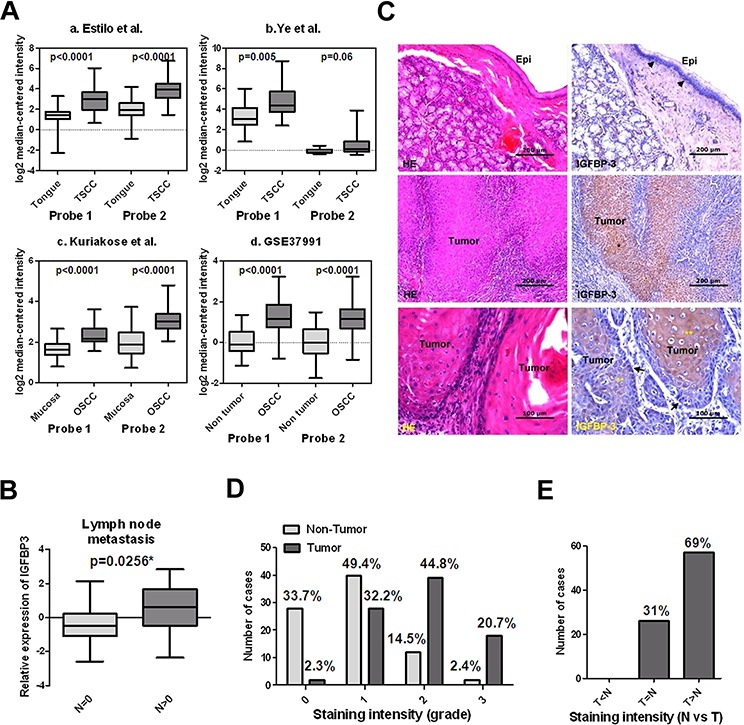
Up-regulated IGFBP3 mRNA and protein in OSCC tissues **A.** Increased IGFBP3 mRNA in oral squamous cell carcinoma (OSCC) in comparison with normal oral tissues by analysis of two probes. Data were obtained from (a) GEO/GSE13601 [28], (b) GEO/GSE6631 [29], (c) GEO/GSE9844 [30] and (d) GEO/GSE37991 [26]. **B.** The mRNA level of IGFBP3 was significantly higher in OSCC tissues with lymph node metastasis than that in OSCC tissues without lymph node metastasis. **C.** Immunohistochemical analysis of IGFBP3 on human OSCC samples. Upper left, H&E staining, showing the non-tumor epithelium (Epi) in a representative OSCC specimen. Upper right, IGFBP3 staining of the non-tumor epithelium, demonstrating cytoplasmic IGFBP3 staining in some basal cells (arrow heads) of the non-tumor epithelium. No apparent IGFBP3 staining was noted on the upper cell layers. Middle left, H&E staining of tumor nests (*) on the same OSCC specimen. Middle right, IGFBP3 staining, showing strong cytoplasmic staining of IGFBP3 on tumor nests compared to their corresponding non-tumor epithelium (bar, 200 μM). Lower left, H&E staining on another OSCC specimen. Lower right, although the less differentiated tumor cells located at the periphery of tumor nests demonstrated some degree of IGFBP3 staining (arrows), strong IGFBP3 staining was especially noted in the center-located, more differentiated tumor cells (**) (bar, 100 μM). **D.** Scoring of IGFBP3 staining intensity in 83 non-tumor epithelium (light grey bar) and 87 tumor specimens (heavy grey bar). 0: no; 1: weak; 2: moderate; 3: strong expression. **E.** Comparison of the IGFBP3 staining intensity between tumor parts (T) and non-tumor epithelium (N) based on each individual histological section.

In the list of highly differentiated expressed genes, we noted IGFBP3, which has been reported to over-express in OSCC [25]. Furthermore, we analyzed IGFBP3 expression using existing cDNA microarray datasets deposited in the publicly available Oncomine database [27]. Having both OSCC and normal tissues, three datasets showed significantly increased expression of IGFBP3 in OSCC when compared with normal tissues (*p* < 0.0001, *p* < 0.001 and *p* < 0.01 in Figure 4A, a-c, respectively), with up-regulation ranging from 2.155 to 3.795-fold according to different datasets and probes [28–30]. Additionally, the expression of IGFBP3 was increased significantly in OSCC samples in comparison with the adjacent noncancerous tissues (*p* < 0.0001 in Figure 4A, d). [26]. Thirty of 40 (75%) OSCC tissue samples exhibited *a* > 1.5-fold increase in IGFBP3 expression relative to their corresponding nontumorous tissues (Figure S2A). Similarly, we performed qRT-PCR to examine IGFBP3 mRNA in 16 OSCC cell lines, observing increased levels of IGFBP3 in all OSCC cell lines when compared with normal human oral keratinocytes (HOK) (Figure S2B). To address the clinical significance of up-regulated IGFBP3 mRNA in OSCC, we observed insignificant correlations with clinicopathological parameters, including pathological stage, and tumor status (Table S2). Importantly, we observed that the level of IGFBP3 mRNA displayed significantly higher in OSCC with lymph node metastasis (*p* = 0.0256, Figure 4B), suggesting the possible role of IGFBP3 in lymph node metastasis.

We next studied the expression patterns of IGFBP3 protein on human OSCC specimens. Figure 4C demonstrated the staining patterns of IGFBP3 in both tumor nests and their corresponding non-tumor epithelia on representative OSCC specimens. Among the 87 specimens available for evaluation of tumor IGFBP3 staining, 65.5% (57/87) of them showed high (score 2, 3) IGFBP3 expression. On the contrary, among the 83 specimens of non-tumor epithelia, only 16.9% (14/83) of them showed high expression of IGFBP3 (*p* < 0.001) (Figure 4D). Additionally, most (69%, 57/83) specimens demonstrated higher IGFBP3 staining intensity in tumor nests as compared to their corresponding non-tumor epithelia (Figure 4E). To further investigated if aberrant expression of IGFBP3 protein is associated with various clinicopathological characteristics in OSCC specimens, we did not observe the significant associations (Table S2). These results suggest that IGFBP3 was frequently up-regulated, both in gene expression and protein levels in OSCC.

### Knockdown of IGFBP3 impairs lymph node metastasis of OSCC

**Figure 5:**
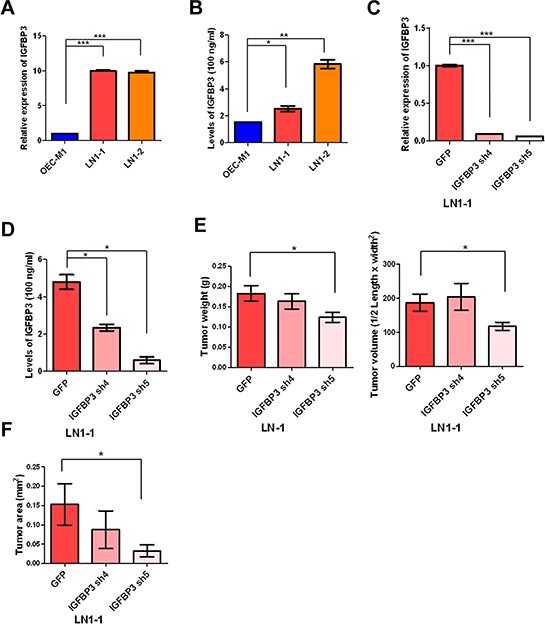
Knockdown of IGFBP3 expression impairs lymph node metastasis **A.** Levels of IGFBP3 mRNA in OEC-M1, LN1–1 and LN1–2 cells were analyzed by qRT-PCR. All amplifications were normalized to an endogenous β-actin control. The relative expression of IGFBP3 mRNA in LN1–1 and -2 cells was normalized to that in OEC-M1 cells. **B.** Levels of IGFBP3 protein in culture supernatant of OEC-M1, LN1–1 and LN1–2 cells were detected by ELISA. **C.** Levels of IGFBP3 mRNA in LN1–1 cells expressing with IGFBP3 shRNA (IGFBP3 sh4 and sh5) and the corresponding controls with lentiviral shRNA against GFP (pLKO-GFP) were determined by qRT-PCR. All amplifications were normalized to an endogenous β-actin control. The relative expression of IGFBP3 mRNA in shRNA expressing cells was normalized to that in the control cells. **D.** Levels of IGFBP3 protein in culture supernatant of LN1–1 cells with IGFBP3 knockdown and the corresponding controls were detected by ELISA. **E.** Quantification of tumor weight (left panel) and volume (right panel) in mice (*n* = 9–10) injected with LN1–1 pLKO-GFP, IGFBP3 sh4 and sh5 cells. **F.** Quantification of tumor area in lymph nodes of mice with orthotopic growth of LN1–1 pLKO-GFP, IGFBP3 sh4 and sh5 cells. The data were expressed as mean tumor area of lymph nodes in mice (*n* = 9–10) bearing orthotopic tumors. Bar, SE; *p < 0.05; **p < 0.01; ***p < 0.001.

To verify the IGFBP3 expression, the results indicated that the levels of IGFBP3 mRNA were up-regulated in LN1–1 and LN1–2 cells in comparison with OEC-M1 cells and showed slightly higher levels of IGFBP3 protein in sublines than the parental cells using qRT-PCR and immunoblot assay, respectively (Figures 5A and S3A). Also, we confirmed the levels of IGFBP3 protein in cultured supernatant from the parental cells and sublines by enzyme-linked immunosorbent assay (ELISA) (Figure 5B). To identify if IGFBP3 is required for tumor growth and lymphatic metastasis, we generated 3 LN1–1 subclones that stably express 2 different IGFBP3 shRNA and one control vector (pLKO-GFP) to access the effects of IGFBP3 silencing in LN1–1 cells. The ablated levels of IGFBP3 mRNA and protein were confirmed by qRT-PCR, ELISA and immunoblot assays (Figures 5C and S3B). We observed that the weight and volume of orthotopic tumors in mice receiving LN1–1 IGFBP3 sh4 and sh5 cells did not consistently decrease when compared with the control tumors (Figure 5E). Importantly, 30 and 40% of the animals had lymph node metastasis after orthotopic injection of LN1–1 IGFBP3 sh4 (*n* = 9) and sh5 (*n* = 10) while 70% of the animals with lymph node metastasis after injection of LN1–1 cells expressing the vector control (Table 1). The average tumor area of lymph nodes was 0.153 ± 0.054, 0.087 ± 0.054 and 0.033 ± 0.016 mm^2^ in mice with control cells, LN1–1 IGFBP3 sh4 and sh5 cells, respectively (Figure 5F). These results suggest that knockdown of IGFBP3 significantly decreased the efficiency of lymphatic metastasis in LN1–1 cells.

### IGF-independent IGFBP3 enhances migration and transendothelial migration of OSCC cells

**Figure 6:**
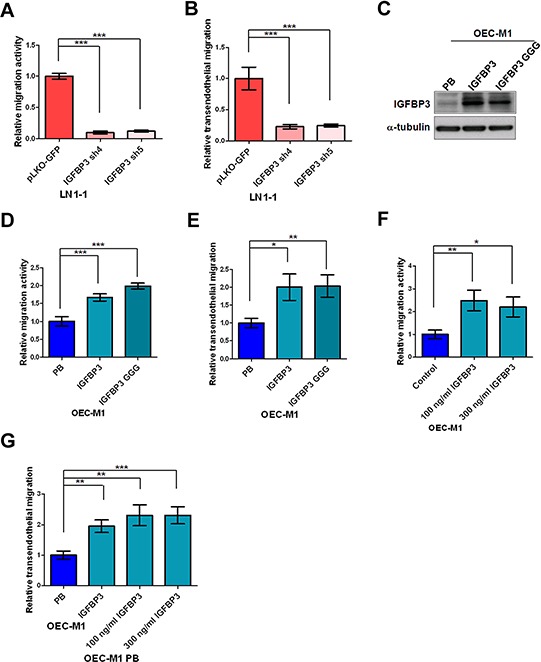
IGF-independent IGFBP3 increases migration and transendothelial migration **A.** Representative data shows the relative activities of migration and **B.** transendothelial migration of LN1–1 cells with IGFBP3 knockdown (IGFBP3 sh4 and sh5) and the corresponding controls (pLKO-GFP). The relative migration/transmigration activity was defined by normalizing the mean of migrated cell/per field in LN1–1 IGFBP3 sh4 or sh5 with that in LN1–1 pLKO-GFP cells. **C.** Immunoblot analysis of IGFBP3 protein in OEC-M1 cells with ectopic wild type and mutant IGFBP3 expression (OEC-M1 IGFBP3, GGG) and vector controls (OEC-M1 PB). α-tubulin serves as an internal control. **D.** Representative data shows the relative activities of migration and **E.** transendothelial migration of OEC-M1 IGFBP3, GGG and OEC-M1 PB cells. The relative migration/transmigration activity was defined by normalizing the mean of migrated cells/per field in OEC-M1 IGFBP3 or GGG cells with that in OEC-M1 PB cells. **F.** Representative data shows the relative migration activity of OEC-M1 cells treated with recombinant IGFBP3. **G.** Representative data shows the relative transmigration activity of OEC-M1 IGFBP3, OEC-M1 PB cells and OEC-M1 PB cells treated with IGFBP3 proteins. The relative migration/transendothelial migration activity was defined by normalizing the mean of migrated cells/per field in OEC-M1 IGFBP3 or IGFBP3 treated cells with that in the control cells. Bar, SE; *p < 0.05; **p < 0.01; ***p < 0.001.

Consistent with *in vivo* tumorigenesis assay, cell proliferation and anchorage-independent growth of LN1–1 cells were not markedly affected by the shRNA-mediated IGFBP3 downregulation (Figure S4). In contrast, IGFBP3 knockdown resulted in a clear reduction in the activities of migration and transendothelial migration (Figure 6A and 6B). The parental OEC-M1 cells were infected with a retroviral vector encoding IGFBP3 or empty vector and ectopic IGFBP3 expression was confirmed by immunoblot assay (Figure 6C). We found that ectopic expression of IGFBP3 in OEC-M1 cells markedly promoted the activities of migration and transendothelial migration (Figure 6D and 6E). To investigate the effects of exogenous IGFBP3, we found that extracellular IGFBP3 did enhance migration of OEC-M1 cells with almost 2-fold increase (Figure 6F). The migration activities of OC3 and SCC9 cells were consistently promoted by exogenous IGFBP3 (Figure S5A). Similarly, transmigration activity of the retroviral vector-expressing OEC-M1 cells was enhanced when incubated with recombinant IGFBP3 proteins (Figure 6G). To investigate if IGF is involved in IGFBP3-induced functions, the parental OEC-M1 cells were expressed with a retroviral vector encoding mutant IGFBP3 (IGFBP3 GGG) with a defect of IGF binding and confirmed by immunoblot assay (Figure 6C). Results demonstrated that ectopic expression of the mutant IGFBP3 did not impair IGFBP3-induced migration and transendothelial migration (Figure 6D and 6E). Again, 37.5 and 42.9% of the experimental animals developed lymph node metastasis after orthotopic injection of OEC-M1 IGFBP3 (*n* = 8) and IGFBP3 GGG (*n* = 8) while 12.5% of the animals with lymph node metastasis after injection of OEC-M1 cells expressing the vector control (OEC-M1 PB) (Table 1). This result suggested that IGFBP3-induced migration, transendothelial migration and lymph node metastasis were involved in an IGF-independent mechanism.

### Silencing of integrin β1 reduces IGFBP3-induced motility and ERK phosphorylation

**Figure 7:**
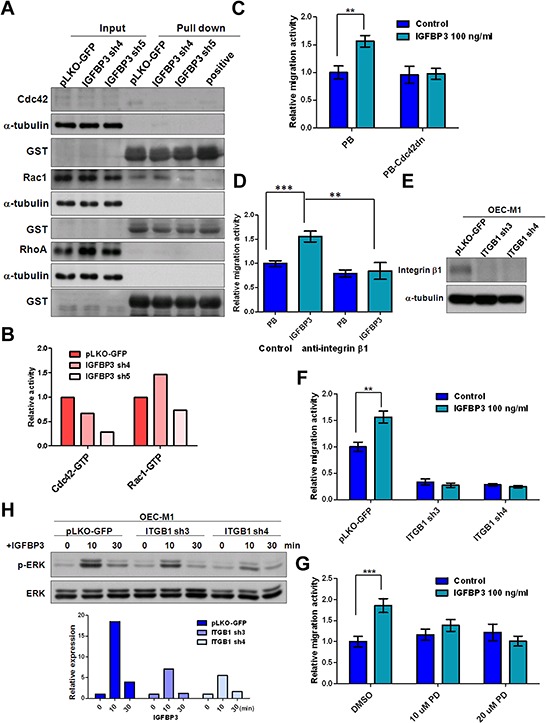
Silencing of integrin β1 inhibits IGFBP3-induced migration **A.** The activities of Cdc42, Rac1 and RhoA were detected in IGBBP3 knockdown cells (IGFBP3 sh4 and sh5) and the corresponding controls (pLKO-GFP). Equal amounts of input protein were subjected to Western blot using anti-Cdc42 (total Cdc42), anti-Rac1 (total Rac1), and anti-RhoA (total RhoA) antibodies. Equal amounts of protein were incubated with GST-PAK1 (detection of active Cdc42 or Rac1) and GST-Rhotekin (detection of active RhoA). Complexes were collected with gluthathione-Sepharose and resolved by Western blot. GTPγS was served as a positive control. Anti-GST antibodies were served as a loading control. **B.** The density of each band was measured by image J and normalized with the controls (LN1–1 pLKO-GFP). The activities of active small GTPase were conducted by dividing the density of active small GTPase to that of GST loading controls. The relative activities were obtained when the activity of active small GTPase in LN1–1 pLKO-GFP were set to 1. **C.** Representative data shows the relative migration activity of OEC-M1 cells with dominant-negative Cdc42 (Cdc42dn) and the corresponding controls (PB). The relative migration activity was defined by normalizing the mean of migrated cell /per field in OEC-M1 PB treated with treated with IGFBP3 and PB-Cdc42dn cells with/without IGFBP3 treatment with that in OEC-M1 PB cells. **D.** Representative data shows the relative migration activity of IGFBP3 expressing cells (OEC-M1 IGFBP3) and the corresponding controls (OEC-M1 PB) with anti-integrin β1 (200 ng/ml) treatment. The relative migration activity was defined by normalizing the mean of migrated cell /per field in OEC-M1 PB and IGFBP3 cells treated with anti-integrin β1 or OEC-M1 IGFBP3 cells treated with IgG antibodies (200 ng/ml) to that in OEC-M1 PB cells treated with IgG antibodies. **E.** Immunoblot analysis of integrin β1 protein in OEC-M1 cells with ITGB1 shRNA expression (OEC-M1 ITGB1 sh3 and sh4) and vector controls (OEC-M1 pLKO-GFP). α-tubulin serves as an internal control. **F.** Representative data shows the relative migration activity of OEC-M1 pLKO-GFP, ITGB1 sh3 and sh4 cells upon IGFBP3 (100 ng/ml) treatment. The relative migration activity was defined by normalizing the mean of migrated cell /per field in OEC-M1 with ITGB1 knockdown and IGFBP3 treatment with that in the control cells. **G.** Representative data showed the relative migration activity of OEC-M1 treated with 10, 20 uM of PD98059 (PD) and dimethyl sulfoxide (DMSO) upon IGFBP3 (100 ng/ml) treatment. The relative migration activity was defined by normalizing the mean of migrated cell/per field in OEC-M1 treated with PD98059 and IGFBP3 with that in the control cells. **H.** Immunoblot analysis revealed knockdown of ITGB1 inhibited IGFBP3-induced ERK phosphorylation at different time points in OEC-M1 cells (upper panel, 0, untreated; 10, 10 min; 30, 30 min for 100 ng/ml IGFBP3 treatment). The ratio of phosphorylated ERK/total ERK was obtained by dividing the intensity of phosphorylated ERK to that of total ERK. The relative expression was obtained when the ration of untreated cells were set to 1 (lower panel). Bar, SE; **p < 0.01; ***p < 0.001.

Small Rho GTPase associated with actin stress fibers are key molecules in the regulation of focal adhesion formation and locomotion in cancer cells [31, 32]. Therefore, we examined whether Rho GTPase activity was associated with IGFBP3 expression via a pull down assay [33, 34]. As compared to the vector control, knockdown of IGFBP3 resulted in the consistent reduction of Cdc42-GTP but not Rac1-GTP in two independent clones (Figure 7A and 7B). IGFBP3 failed to induce cell migration in OEC-M1 cells expressing dominant-negative Cdc42 (Figure 7C), suggesting that the activation of Cdc42 is involved in IGFBP3-mediated migration. Based on the previous studies showing that small GTPase is the downstream effectors of integrin–mediated cell migration, we further explore whether IGFBP3-induced migration was mediated by integrin β1, which is interacting with IGFBP3 as shown previously [35–39]. The neutralized antibodies of integrin β1 were found to abolish IGFBP3-induced migration when compared to the IGFBP3 expressing cells treated with IgG antibodies (Figure 7D). Additionally, we generated 3 OEC-M1 subclones that stably express 2 different ITGB1 shRNA and one control vector to decide the effects of integrin β1 silencing in IGFBP3-induced migration. The abolished levels of integrin β1 protein were confirmed by immunoblot assays (Figure 7E). Results indicated that ITGB1 knockdown resulted in a significant reduction in the migration activity of OEC-M1 cells. Importantly, exogenous IGFBP3 hardly induced cell migration in OEC-M1 cells with low expression of integrin β1. Similarly, IGFBP3-induced migration was abolished in OC3 cells with ITGB1 knockdown (Figure S5B–S5C). After examining the IGFBP3-induced signaling, we found that ERK phosphorylation was induced upon IGFBP3 stimulation (Figure S6). Treatment of PD98059, an inhibitor of mitogen-activated protein kinase kinase (MEK), reduced IGFBP3-induced migration in OEC-M1 cells (Figure 7G). Therefore, by checking the phosphorylated ERK in ITGB1 knockdown cells, the level of phosphorylated ERK was decreased in ITGB1 knockdown cells stimulated by IGFBP3 (Figure 7H). Take together, our data demonstrated that integrin β1 is required for IGFBP3-induced motility and ERK phosphorylation.

## DISCUSSION

The traditional subcutaneous transplantation process is not an ideal model for studying the underlying mechanisms of tumor metastasis due to the limited spontaneous metastasis. Several studies has reported that when oral tumor cells were orthotopically inoculated into tongues, mouth floor and axilla of nude mice, tumor formation, lymph node metastasis and lung metastasis were developed [40–43]. In an attempt to isolate highly metastatic OSCC cells, we failed to observe the cervical lymph node metastasis in animals by tongue injecting due to early sacrifice of animals with difficulty in eating. By inoculating OSCC cells into mucosa of the oral cavity, we have established an animal model of cervical lymph node metastasis and isolated the highly metastatic sublines from poorly metastatic OSCC cells by *in vivo* selection (Table 1). Matsui et al. and Morita et al. obtained highly metastatic tumor cells after three repeated selections through inoculating OSCC cells into the tongues of nude mice, followed by isolating tumor cells from the cervical lymph nodes [43, 44]. Our major concerns were that the rates of lymph node metastasis in LN1–1 and LN1–2 sublines were markedly high (70–83%) and that the biological characteristics of these selected cells would be altered if more passages and subculture were performed (Table 1).

Similar to the previous studies, our results suggest that the metastatic capacity of the lymph nodes and tumor growth were specifically increased in sublines by *in vivo* selection (Figure 2 and 3) [41, 43, 45]. In contrast, the obtained sublines did not show much greater property of migration (Figure 1D) [44]. Interestingly, we observed that LN1–1 and LN1–2 sublines showed greater ability of transendothelial migration than their parental cells (Figure 1E). The results indicated that direct contact with lymphatic endothelial cells (or their extracellular matrix) is required to reveal their enhanced transmigration properties [46].

Gene ontology analysis with IPA, comparing differentially expressed genes between OEC-M1 and LN1–1 or LN1–2 subline, showing that leukocyte extravasation signaling (*p* = 4.86E-04) pathway was scored significantly (ratio = 13/210, 0.062 in Figure S1B). This suggests that the expression of genes that are involved in leukocyte trafficking have been implicated in transendothelial migration and cancer cell extravasation might be selected in OSCC sublines. Matrix metalloproteinases (MMPs) are highly involved in degradation of extracellular matrix, a key process of cancer metastasis [47], MMP2 and MMP9 are the principal enzyme involved and reported to be overexpressed in OSCC [48]. According to our microarray data, we found that MMP1 and MMP10 were highly expressed in two sublines as compared to the parental cells. This result was further confirmed by qRT-PCR. The secreted MMP1 and MMP10 proteins were determined by Western blot using collected conditioned medium from OEC-M1, LN1–1 and LN1–2 cells. The high levels of secreted MMP1 and MMP10 were detected in OSCC cells with high potential of lymphatic metastasis (Figure S7), indicating the roles of MMP1 and MMP10 in lymph node metastasis of OSCC as shown previously [49, 50]. Furthermore, comparison with the previously published cDNA microarray studies for lymphatic metastasis in OSCC, we did not find any common gene that is reportedly associated with lymph node metastasis in our gene lists with highly de-regulated expression [49, 51–56]. This, however, might be caused by the specific epidemiology. Similarly, bone marrow stroma cell antigen 2 (*BST2*), one of the up-regulated genes in Table 2 is recently identified as an independent prognostic factor of patient survival and associated with positive N stage in Taiwan OSCC patients [57]. Based on the datasets from Oncomine and Taiwan OSCC patients, we observed that the level of IGFBP3 mRNA was up-regulated in tumorous tissues and associated with the N stage of clinicopathological features, suggesting the role of IGFBP3 in lymph node metastasis of OSCC (Figure 4B). Additionally, we observed up-regulated IGFBP3 protein in OSCC tissues as compared to the non-tumor epithelium using IHC (Figure 4C–4E). Given that IGFBP3 is a secretary protein, we considered whether IGFBP3 protein could be measured in the plasma of OSCC patients and used to differentiate the status of lymph node metastasis. By ELISA, we found that IGFBP3 protein could be detected in plasma of OSCC patients as shown in the previous studies [58], however, the level of IGFBP3 protein did not demonstrate any difference between OSCC with or without lymph node metastasis (Figure S8). The differential expression pattern was not observed for IGFBP3 protein, possibly because it is also secreted by additional cell types (e.g. liver tissues), and thereby potentially masking the signal emanating from the tumor itself.

Our study first demonstrated that IGFBP3 is sufficient and required for migration, transendothelial migration and lymph node metastasis of OSCC cells as evidenced that knockdown of IGFBP3 reduced and ectopic expression of IGFBP enhanced the activities (Figure 5 and 6). This data provides the evidence for high expression of IGFBP3 in OSCC with lymph node metastasis, as previously reported [25]. Furthermore, by experiments using mutant IGFBP3 GGG that cannot bind IGF, we found that IGFBP3 enhanced the migration, transmigration and lymph node metastasis of OSCC cells in an IGF-independent mechanisms (Figure 6D–6E and Table 1). Although it is clear that IGFBP3 achieves biological effects independent of the IGF/IGF1R axis, the mechanisms by which these effects are achieved are still not entirely understood.

As shown in the previous studies [38, 59], the activity of small GTPase is also involved in IGFBP3-induced migration as evidenced by the result that the reduced active Cdc42 was detected in IGFBP3 knockdown cells and IGFBP3-induced migration was destroyed in dominant-negative Cdc42 expressing cells (Figure 7A–7C). In our study, knockdown of integrin β1 or neutralized antibodies did not sustain the IGFBP3-induced migration activity and ERK phosphorylation, suggesting that the blockage of integrin β1 impaired IGFBP3-mediaetd functions (Figure 7D–7G). Similar to our data, several studies demonstrated that the effects of C-terminal metal-binding domain (MBD) of IGFBP3, showing a direct physical interaction with integrins or IGFBP3-promoted functions were inhibited by antibodies to integrins [39, 60]. Thus, the detail for relationships between IGFBP3 and integrin β1 needs further investigation. Moreover, the activated integrin-ERK pathway has been shown to induce proliferation, migration and invasion by malignant cells in response to external stimuli [61–64]. Ahmed et al. reported that the physical interaction between integrin β6 and ERK2 defines anther paradigm of integrin/ERK mediated signaling. However, this direct link was not detected between integrin β1 and ERK2 [65]. In our study, treatment of PD98059 reduced IGFBP3-induced migration in OEC-M1 cells (Figure 7G). The data suggested that the ERK activation by the upstream activating kinase MEK was critical for IGFBP3-induced motility. By Western blot, IGFBP3-induced ERK phosphorylation was reduced in ITGB1 knockdown cells, suggesting the IGFBP3-integrin β1-ERK phosphorylation axis in regulation of OSCC cell motility (Figure 7H).

Collectively, the data presented herein demonstrate that the expression of IGFBP3 is up-regulated in highly metastatic OSCC sublines developed via *in vivo* selection and OSCC tissues. IGFBP3 acts in to an IGF-independent manner to enhance *in vitro* migration/transmigration as well as lymph node metastasis of OSCC cells. Interestingly, silencing of integrin β1 impairs IGFBP3-enhanced motility and ERK activation, indicating that integrin β1 is critical in IGF-independent IGFBP3-mediated functions.

## MATERIALS AND METHODS

### Ethics statement

All animal studies were performed in strict accordance with the recommendations in the guidelines for the Care and Use of Laboratory Animals of National Health Research Institutes, Taiwan. The protocols were approved by the Institutional Animal Care and Use Committee of National Health Research Institutes (Protocol No: NHRI-IACUC-100047-A and NHRI-IACUC-100136-A).

### *In vivo* section by orthotopic injection in nude mice

The cells in logarithmic phase were digested with trypsin (Invitrogen) and collected. The density of collected cells was adjusted to 1 × 10^7^/ml with sterilized phosphate buffered saline (PBS, Invitrogen). The viable cell count was over 95% as measured by trypan blue staining (Invitrogen). Male nude mice (BALB/cAnN.Cg-Foxn1^nu^/CrlNarl) with 5–6 weeks of age were purchased from the National Laboratory Animal Center (NALC) and anesthetized via inhalation of 5% Isoflurane (Piramal Critical Care). After successful anesthesia, 50 μl of the cell suspension was injected into the buccal mucosa of mice and carefully monitored. For *in vivo* selection, the cervical lymph nodes from sacrificed mice were excised on a clean bench and cut into pieces on day 42 and 56 after the oral injection. The cells were collected with PBS, centrifuged and cultured in RPMI medium with 10% FBS at 37°C in an incubator containing 5% CO_2_. When the cell confluence reached 80%, cells were passaged for further culture. After repeated passages, stably growing tumor cells were obtained.

### Pathological exam

The paraffin-embedded tissue blocks were stained by conventional hematoxylin and eosin (H&E, Sigma, St Louis, MO, USA) staining and observed under a light microscope.

### Morphological analysis

Exponentially growing cells were observed via inverted phase-contrast microscope as previously described [66]. Cultured cells on slips were fixed and followed the protocols as previous described [67]. After stained with Alex Fluo 488 phalloidin (Molecular Probes, Ungene, OR, USA), the slips were mounted and viewed under a fluorescence confocal microscope.

### Cell proliferation

The proliferation curves were determined by calculating the mean value of absorbance measurement at 490 nm using a 96-well plate reader as described previously [68].

### Soft agar assay

Soft agar assay was performed as previously described [68].

### Cell migration

Migration assays were performed using transwells as described previously [67]. The neutralized anti-integrin β1 and anti-IgG antibodies were purchased from Millipore (MAB2253 and PP100, respectively; EMD millipore, Billerica, MA, USA). The PD98059 and dimethyl sulfoxide (DMSO) were obtained from Sigma.

### Transendothelial migration assay

Human lymphatic endothelial cells (LECs), purchased from PromoCell (Heidelberg, Germany) were seeded at 2–2.5 × 10^5^ cells in 24-well transwell inserts with a pore size of 8 μm (BD, Franklin Lakes, NJ, USA) to grow to confluence. Cells (10^5^ cells/ml) were labeled using PKH26 Red Fluorescent cell linker kits (Sigma) according to the instructions, suspended in serum-free medium and then added to the endothelial monolayer. After 20–24 hours incubation, the migrated cells on the bottom face of the insert were counted under the fluorescent microscope. The transmigrated cells were measured in 5 random fields/per insert at the 100 × magnification.

### DNA extraction and amplification of human short tandem repeat sequences with PCR

Genomic DNA was prepared using a genomic DNA extraction kit according to the manufacturer's instructions (Qiagen, Valencia, CA, USA). DNA yield and purity was assessed by absorption measurement at 260 nm and by determination of the 260/280 ratio using a Nanodrop 1000 spectrophotometer (NanoDrop Technologies, Wilmington, DE, USA). Sequences for the ten primers used in this study were published with modifications as shown at STRbase [69, 70]. Amplification was performed in a 10 μl final volume using reaction conditions recommended by the manufacturer's instructions (Toyobo, Osaka, Japan). Cycling was performed on a thermal cycler using the cycling profile: An initial 94°C 2 min, 98°C 10 sec, 58°C for 30 sec, 68°C for 1 min, for 35 cycles, and finally soak in 4° C. Capillary electrophoresis was performed on a 3700 Genetic Analyzer (Applied Biosystems; Foster City, CA). Allele designation was derived by comparing sample peaks by allelic ladder peaks using the Gene Mapper 3.5 software (Applied Biosystems).

### Microarray and hybridization processing of expression data

Targets were synthesized, amplified, labeled, and purified using the TargetAmp Nano-G Bioti-aRNA Labeling kit (Epicentre, Madison, WI) according to the manufacturer's instructions. Briefly, 500 ng of total RNA were reversed transcribed and subject to second strand cDNA synthesis, followed by *in vitro* transcription and complementary RNA labeling with biotin-dUTP. Labeled targets were then subjected to hybridization to Illumina BeadChips Human HT12 according to the protocol recommended by Illumina (Illumina, San Diego, CA, USA). The chips were scanned on the Illumina BeadArray 500GX reader and images were processed by Illumina BeadScan software. Genome Studio (Illumina) and Partek Genomics Suit (Partek, St. Louis, MI, USA) software packages were used for preliminary data analysis. A model-based background correction method was used to correct the background noise and to normalize the data before analysis to obtain differentially expressed genes in testing samples [71]. Microarray data are available in the Gene Expression Omnibus (GEO) under Accession No GSE62326. Genes with less than raw signal <100 were filtered out, leaving genes for further analysis.

### Data analysis

Differentially expressed genes were selected based on expression fold change greater than 1.5 fold and significance *p* value < 0.05. Ingenuity Pathways analysis (IPA, Ingenuity Systems, Redwood City, CA, USA) was used to gain a overview of the general biology that is associated with microarray data. In IPA, Fisher's exact was used to calculate a *p* value determining the probability that each bio-function assigned to that dataset is due to chance alone. Canonical pathway analysis tool in IPA was used to identify signaling and metabolic pathways associated with the database. Genes from the dataset that met the fold change cut-off of 1.5 (*p* < 0.05) were considered for the further analysis. The significance of the association between the dataset and the canonical pathways was measured according to either fold change or *p* value.

### Oral squamous cell carcinoma cell lines

Human oral keratinocytes (HOK) were purchased from ScienCell Research Laboratories and cultured in an oral keratinocyte medium (OKM; ScienCell Research Laboratories, Carlsbad, CA, USA) according to the manufacturer's instructions. OSCC cell lines, including CGHNC9, OC3, OEC-M1, TW2.6, FaDu, KB, SCC4, SCC15, SCC9, SCC25, UT-MUC-1, YD-15, DOK, Tu183, UMSCC1 and HSC3, were cultured at 37°C in a 5% CO_2_ atmosphere within 3 months of resuscitation from the frozen aliquots, with lower than 20 passages in each experiment as described previously [72].

### Immunohistochemistry

The immunohistochemistry (IHC) was performed as described previously [66]. Primary antibodies were used as follows: anti-Ki-67 (NCL-Ki67p, Novacastra Laboratories, Newcastle upon Tyne, UK) or anti-LYVE (07–358, Upstate Biotechnology, Lake Placid, NY, USA). Sections were counterstained with hematoxylin and viewed under light-field microscope. Using the imaging software (ImmunoRatio), the percentage of positive Ki-67 stain was defined as the total intensity of positive nuclei of tumor cells divided by that of the total nuclei in the field (original magnification 400 ×) [73]. For detection of IGFBP3 expression, formalin-fixed, paraffin-embedded OSCC specimens containing both tumor nests and adjacent non-tumor epithelium were obtained from Department of Pathology at National Cheng Kung University Hospital. Briefly, 4 μm thickness slides were deparaffinized and rehydrated through graded alcohols and rinsed with phosphate-buffered saline solution. No antigen retrieval procedure was performed. After blocking endogenous peroxidase activity, sections were then incubated with mouse anti-human IGFBP3 (MAB305, 1:50, R&D, Minneapolis, MN, USA) at 4°C overnight. After wash, sections were then incubated with goat anti-mouse secondary antibodies conjugated with horseradish peroxidase (HRP) polymer (Biocare, Concord, CA, USA) at room temperature for 30 min. Specific signals were then developed using diaminobenzidine (Biocare) as chromogen. Mouse IgG_2B_ was used as isotype control. Sections were then counterstained with hematoxylin and observed under light microscope. The level of IGFBP3 on each specimen was scored as 0, 1, 2, 3 (0 = negative, 1 = weak, 2 = intermediate and 3 = strong) according to its staining intensity. For each specimen, the tumor part and the adjacent non-tumor epithelial part were scored separately.

### Plasmids

RNAi-mediated depletion was achieved by infecting cells with pLKO-based lentiviruses encoding short hairpin RNA (shRNA) targeting specific mRNA (National RNAi Core Facility/Academia Sinica, Taipei, Taiwan). Knockdown clones for IGFBP3 and integrin β1 (ITGB1) were as follows: IGFBP3 sh4 (TRCN000072511), IGFBP3 sh5 (TRCN0000072512), ITGB1 sh3 (TRCN0000029647) and ITGB1 sh4 (TRCN0000029645). Wild type and mutant IGFBP3 cDNA from Dr. Hiroshi Nakagawa and dominant-negative Cdc42 cDNA from Dr. Lui-Hai Wang were constructed in pBabe-puro vector [74]. Expression constructs were stably expressed in targets cells as described previously [66].

### Quantitative reverse transcription-polymerase chain reaction (qRT-PCR)

The qRT-PCR was performed as described previously [66]. The primer sequences used are listed below. IGFBP3-F: 5′ CAAGCGGGAGACAGAATATGG; IGFBP3-R: 5′ GGACTCAGCACATTGAGGAACTT; β-actin-F: 5′ TGGATCAGCAAGCAGGAGTATG; β-actin-R: 5′ GCATTGCGGTGGACGAT. All amplifications were performed in triplicate.

### Immunoblot assay

Immunoblot assay was performed as previously described [66]. Conditioned medium was collected and concentrated by Amicon Ultra-4 Centrifugal Filters Ultracel-30K (Merck Millipore). The blots were stained with InstantBlue (Expedeon Inc. San Diego, CA, USA). Primary antibodies were used as follows: anti-IGFBP3 (MAB305, R&D Systems), anti-integrin β1 (Santa Cruz, Dallas, TX, USA), anti-α-tubulin (MS-581-P0, Thermo Scientific), anti-focal adhesion kinase (FAK, sc-557, Santa Cruz), anti-phosphorylated FAK (611806, BD), anti-Src (#2109, Cell Signaling, Beverly, MA, USA), anti-phosphorylated Src (05–677, Millipore), anti-nuclear factor kappa-light-chain-enhancer of activated B cells (NF-kB, sc-8008, Santa Cruz), anti-phosphorylated NF-kB (#3033, Cell Signaling), anti-protein kinase B/AKT (#9272, Cell Signaling), anti-phosphorylated AKT (#9271, Cell Signaling), anti-ERK (sc-94, Santa Cruz). anti-phospho-ERK (sc-7383, Santa Cruz), anti-integrin-linked kinase (ILK, GTX101691, GeneTex, Irvine, CA, USA), MMP1 (RB1536P0, Thermo Scientific) and MMP10 (AP6194a, Abgent, San Diego, CA, USA).

### Enzyme-linked immunosorbemt assay (ELISA)

This study was approved by the institutional review boards of the National Cheng Kung University Hospital and the National Health Research Institutes. A signed informed consent was obtained from every study participant. Recruitment of OSCC patients was conducted in the Department of Otolaryngology at the National Cheng Kung University Hospital from September 1, 2010 to October 31, 2013. Blood samples were collected at diagnosis from 90 OSCC patients in heparin-containing phlebotomy tubes. Plasma was obtained following the phlebotomy by centrifugation at 3000 × g for 30 min at 4°C. The clear plasma supernatant was stored at −80°C until use. Clinical parameters, such as TMN stage were retrospectively collected by reviewing patients' charts. Following the manufacturer's instructions, human IGFBP3 protein levels were quantified using Quantikine IGFBP3 ELISA kit (R&D Systems).

### GTPase activity assay

GTPase activity assay was conducted as described previously [59]. The amount of total input and glutathione-S-transferase (GST)-Rhotekin-, GST-PAK1–bound small GTPase was determined by Western blot with anti-RhoA (Millipore), anti-Cdc42 (Millipore) and anti-Rac1 antibody (Novous Biologicals, Littleton, CO, USA), respectively. GTPγS (Millipore) was served as a positive control to maintain the active form of GTPase in the reaction. GST detection by anti-GST antibody (Santa Cruz) was served as a loading control. The protein intensity was measured and quantified by Image J (National Institutes of Health, Bethesda, MD, USA).

### Statistical analysis

Data were expressed as mean ± standard error of the mean (SE) and analyzed as described previously [72]. For all comparisons, *p* < 0.05 was considered statistically significant.

## SUPPLEMENTARY TABLES AND FIGURES



## Abbreviations

OSCC: oral squamous cell carcinoma
IGFBP3: insulin-like growth factor binding protein 3
IGF: insulin-like growth factor
ERK: extracellular signal-regulated kinase
LYVE-1: lymphatic vessel endothelial hyaluronic acid receptor 1
qRT-PCR: quantitative reverse transcription-polymerase chain reaction
IHC: immunohistochemistry
H&E: Hematoxylin and Eosin
