# Correction: Insulin-like growth factor-independent insulin-like growth factor binding protein 3 promotes cell migration and lymph node metastasis of oral squamous cell carcinoma cells by requirement of integrin β1

**DOI:** 10.18632/oncotarget.9711

**Published:** 2016-05-30

**Authors:** Yi-Chen Yen, Jenn-Ren Hsiao, Shih Sheng Jiang, Jeffrey S. Chang, Ssu-Han Wang, Ying-Ying Shen, Chung-Hsing Chen, I-Shou Chang, Jang-Yang Chang, Ya-Wen Chen

Present: During final preparation of the manuscript by the authors, OAS1 (2′-5′- oligoadenylate synthetase 1) was mixed up with OSA1 (ARID1B AT rich interactive domain 1A). Additinally, OAS2 (2′-5′- oligoadenylate synthetase 2) was mixed with OSA2 (ARID1B AT rich interactive domain 1B). These errors resulted in incorrect presentation of Table 2.

Corrected: Correct Table 2 is provided below. The authors sincerely apologize for this error.

Original article: Oncotarget. 2015; 6(39): 41837-55. doi: 10.18632/oncotarget.5995.

**Table 2:** List of the top 10 up-regulated and top 5 down-regulated genes

De-regulated gene	Description	Fold change
IFI27	Interferon, alpha-inducible protein 27	162
OAS2	2′-5′-oligoadenylate synthetase 2	21
DHX58	DEXH (Asp-Glu-X-His) box polypeptide 58	10
CFB	Complement factor B	10
ANKRD38	KN motif and ankyrin repeat domains 4	7.2
PDZK1	PDZ domain containing 1	6.6
OAS1	2′-5′-oligoadenylate synthetase 1	6.5
BST2	Bone marrow stromal cell antigen 2	6.4
IGFBP3	Insulin-like growth factor binding protein 3	5.5
IFI6	Interferon, alpha-inducible protein 6	5.1
PKIA	Protein kinase (cAMP-dependent, catalytic) inhibitor alpha	0.38
SLC47A1	Solute carrier family 47 (multidrug and toxin extrusion), member 1	0.41
CGNL1	Cingulin-like 1	0.45
EPHX1	Epoxide hydrolase 1, microsomal (xenobiotic)	0.48
PGD	Phosphogluconate dehydrogenase	0.53

