# Supplementary material for: Insulin-like growth factor-independent insulin-like growth factor binding protein 3 promotes cell migration and lymph node metastasis of oral squamous cell carcinoma cells by requirement of integrin β1
Source: Oncotarget. 2015 Oct 19;6(39):41837–55. doi: 10.18632/oncotarget.5995 (PMC4747192; doi:10.18632/oncotarget.5995)
Supplement: Supplementary file 1 [file oncotarget-06-41837-s001.pdf]

SUPPLEMENTARY TABLES AND FIGURES

Supplementary Table S1: Short tandem repeat (STR) profiles of three oral squamous cell carcinoma (OSCC) cell lines

Markers (bp/ peak number)	OEC-M1		LN1–1		LN1–2	
TH01	194.31/9		194.41/9		194.32/9	
CSF1PO	313.68/12		313.66/12		313.67/12	
D13S317	185.08/10		185.23/10		185.09/10	
D16S539	279.29/10	287.24/12	279.47/10	287.73/12	279.34/10	287.28/12
VWA	136.87/14	145.89/16	137.07/14	146.16/16	136.80/14	145.94/16
TPOX	229.45/8		229.49/8		229.42/8	
D5S818	131.59/11		131.52/11		131.59/11	
D7S820	221.64/8	233.49/11	221.82/8	233.73/11	221.74/8	233.57/11
D3S1358	123.33/15	135.73/18	123.38/15	135.74/18	123.35/15	135.69/18
Amel	211.05/X	216.53/Y	210.92/X	216.52/Y	211.06/X	216.58/Y

Supplementary Table S2: Correlations between clinical parameters and relative IGFBP3 mRNA and cancerous IGFBP3 protein in oral squamous cell carcinoma (OSCC) patients[#]

Clinical features	Case no	Relative IGFBP3 mRNA	*p*-value	Case no	Cancerous protein score	*p*-value
TNM stage						
Stage I-II	15	2.259 ± 0.496	0.0737	27	1.889 ± 0.1343	0.6903
Stage III-IV	25	4.491 ± 0.8878		60	1.817 ± 0.1049	
T classification						
T1–2	23	2.726 ± 0.5599	0.0739	44	1.773 ± 0.117	0.4228
T3–4	17	4.910 ± 1.16		43	1.907 ± 0.1191	
N classification						
N = 0	23	2.508 ± 0.5148	0.0256*	51	1.922 ± 0.108	0.24
N = 1–2	17	5.205 ± 1.16		36	1.722 ± 0.13	
Lymphovascular invasion						
No	21	2.706 ± 0.5559	0.0703	47	1.809 ± 0.1123	0.5083
Yes	18	4.936 ± 1.119		38	1.921 ± 0.1272	

[#]all of these 40 patients were male patients without any evidence of distant metastasis disease prior to surgery (M0). None of them had N3 disease.
[*]$p < 0.05$ by student *t* test

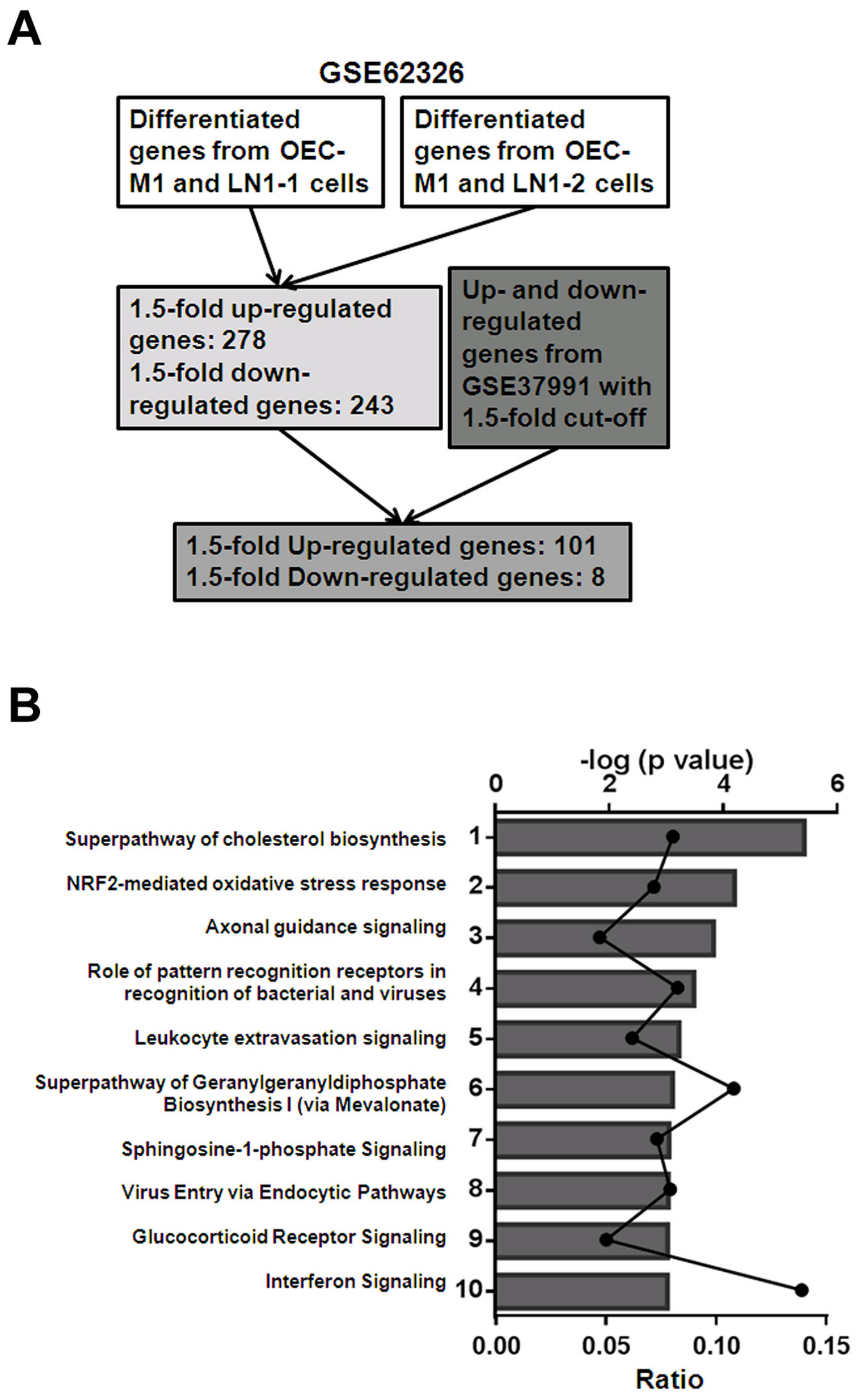

Supplementary Figure S1: Microarray analysis of de-regulated genes among the three OSCC cells. A. The scheme shows the data process after microarray analysis. **B.** Significant canonical pathways were ranked by negative log of the calculated hypergeometric *p* value. The curve represented the ratio of genes in the pathways.

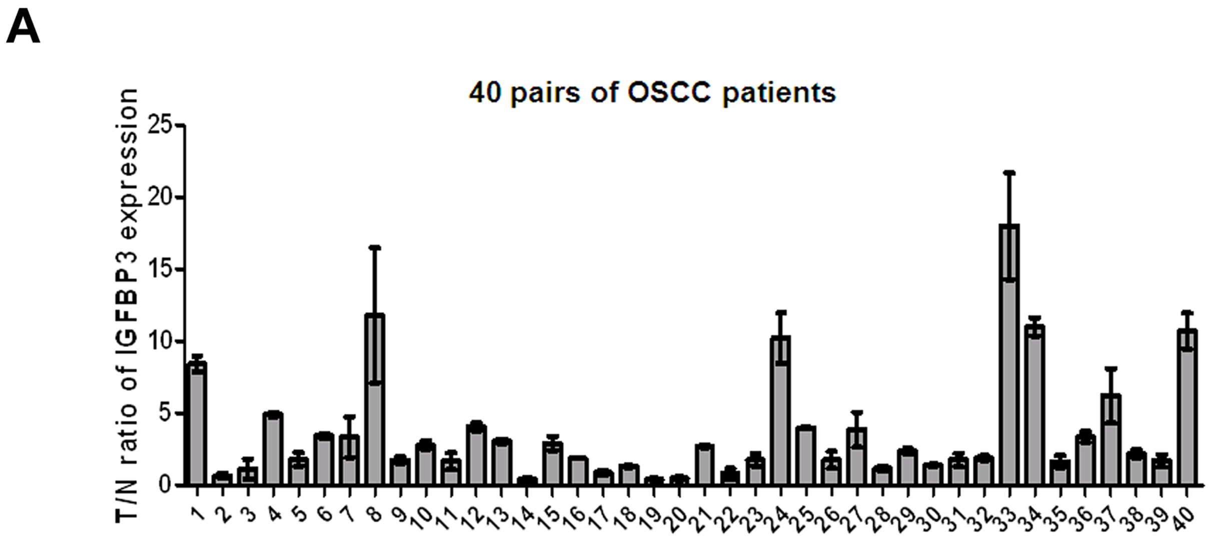

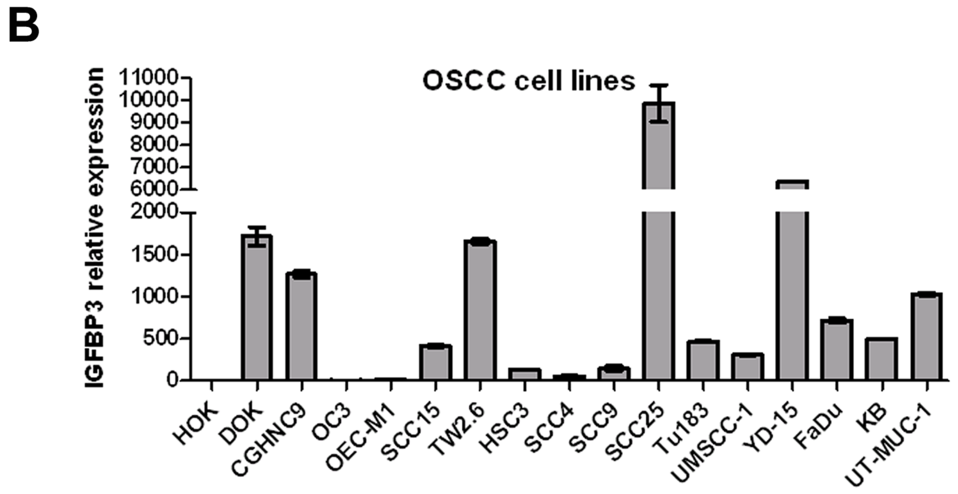

Supplementary Figure S2: Expression of IGFBP3 mRNA in OSCC tissues and cell lines. A. The data revealed significant up-regulation of IGFBP3 mRNA in 30/40 (75%) OSCC tissues, with 1.5-fold increase than the corresponding nontumorous tissues. The relative IGFBP3 expression was determined by dividing the detected signal from a tumorous tissue by that from its corresponding nontumorous tissues. The data were shown by averaging two different probes from the dataset GSE37991. **B.** The levels of IGFBP3 mRNA in 16 OSCC cell lines were analyzed by qRT-PCR. All amplifications were normalized to an endogenous β-actin control. The relative expression of IGFBP3 mRNA in OSCC cells was normalized to that in human oral keratinocytes (HOK) cells.

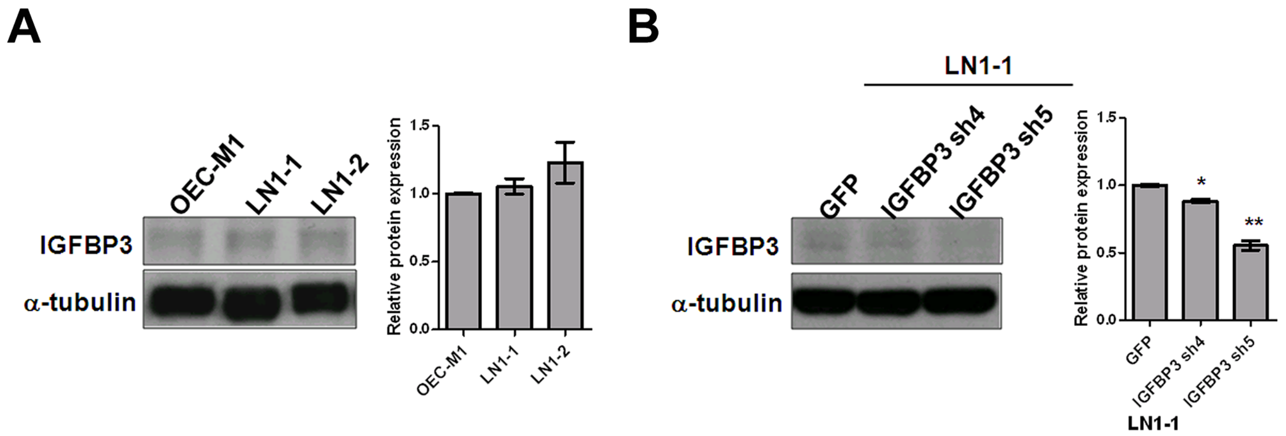

Supplementary Figure S3: Detection of IGFBP3 protein by immunoblot assay. A. Immunoblot analysis of IGFBP3 protein in OEC-M1, LN1–1 and LN1–2 cells. α-tubulin serves as an internal control. Ratios were determined by dividing the normalized protein levels in sublines with that in OEC-M1 cells. **B.** Immunoblot analysis of IGFBP3 protein in LN1–1 cells with IGFBP3 knockdown (IGFBP3 sh4 and sh5) and the corresponding controls (pLKO-GFP).Ratios were determined by dividing the normalized protein levels in LN1–1 IGFBP3 sh4 and sh5 cells with that in LN1–1 pLKO-GFP cells. Bar, SE; *$p < 0.05$; **$p < 0.01$.

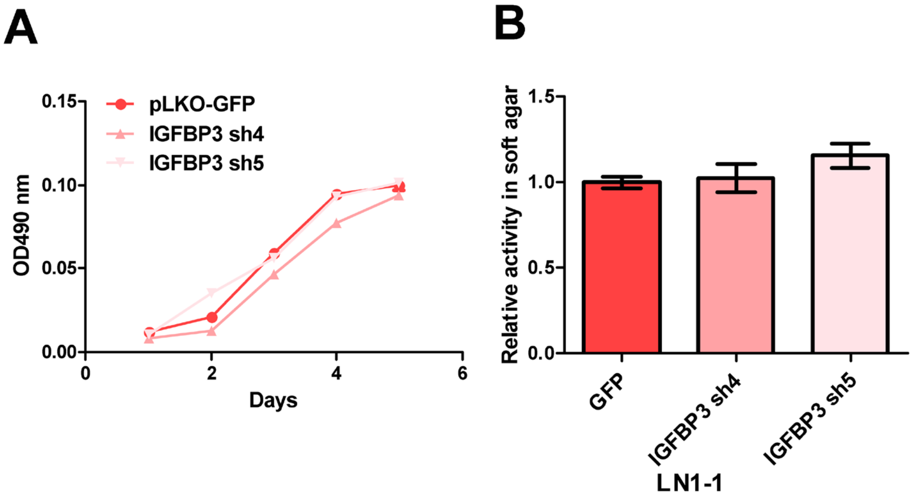

Supplementary Figure S4: Knockdown of IGFBP3 has no effects on cell proliferation and anchorage-independent growth. **A.** Representative data show cell proliferation in LN1–1 cells with IGFBP3 knockdown (IGFBP3 sh4 and sh5) and their corresponding control cells (pLKO-GFP). **B.** Representative data demonstrate anchorage-independent growth activity for LN1–1 pLKO-GFP, IGFBP3 sh4 and sh5 cells. The relative activity was determined by normalizing the mean of colonies/per plate in LN1–1 IGFBP3 sh4 and sh5 to that in LN1–1 pLKO-GFP cells. Bar, SE.

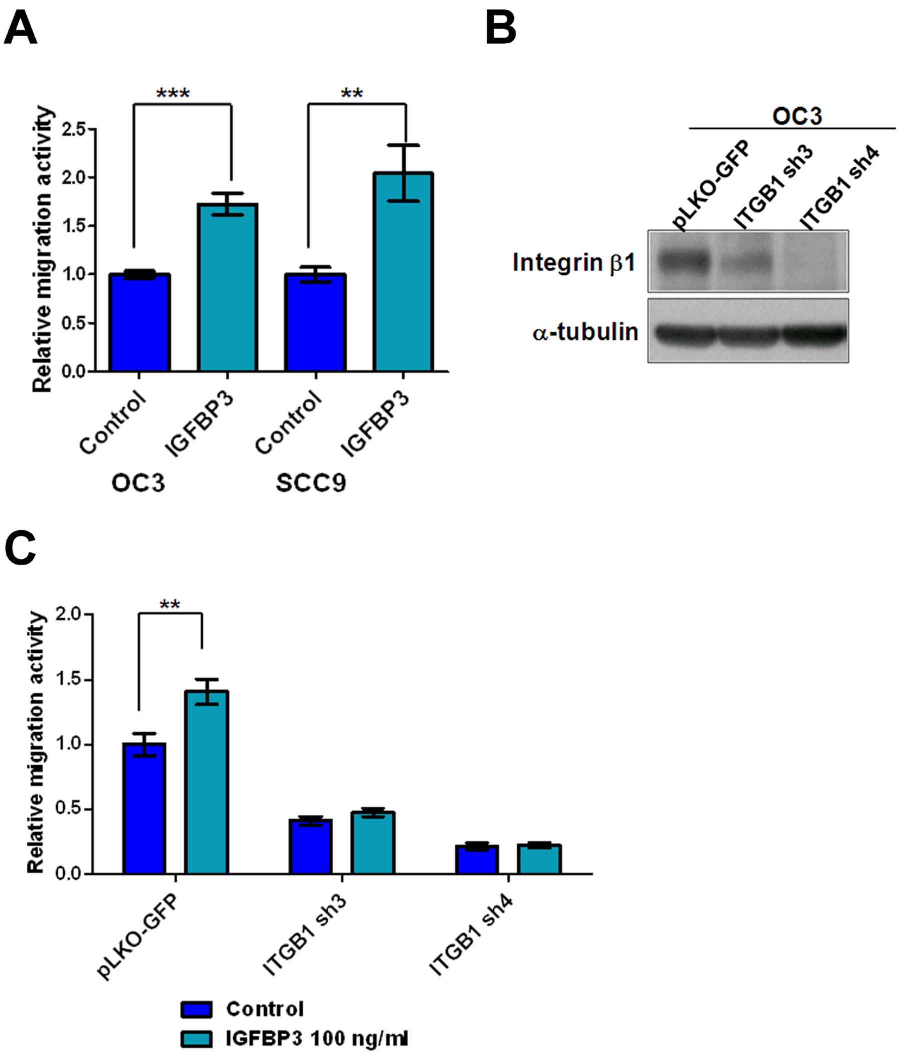

Supplementary Figure S5: Intergrin β1 is required for IGFBP3-indcued migration in OC3 cells. A. Representative data show the relative migration activity of OC3 and SCC9 cells treated with recombinant IGFBP3. The relative migration activity was defined by normalizing the mean of migrated cells/per field in IGFBP3-treated cells with that in the untreated cells. **B.** Immunoblot analysis of integrin β1 protein in OC3 cells with ITGB1 shRNA expression (OC3 ITGB1 sh3 and sh4) and vector controls (OC3 pLKO-GFP). α-tubulin serves as an internal control. **C.** Representative data shows the relative migration activity of OC3 pLKO-GFP, ITGB1 sh3 and sh4 cells upon 100 ng/ml IGFBP3 treatment. The relative migration activity was defined by normalizing the mean of migrated cell /per field in OC3 pLKO-GFP cells treated with IGFBP3 and OC3 ITGB1 sh3 and sh4 with/without IGFBP3 treatment with that in untreated OC3 pLKO-GFP cells. Bar, SE; ***p* < 0.01; ****p* < 0.001.

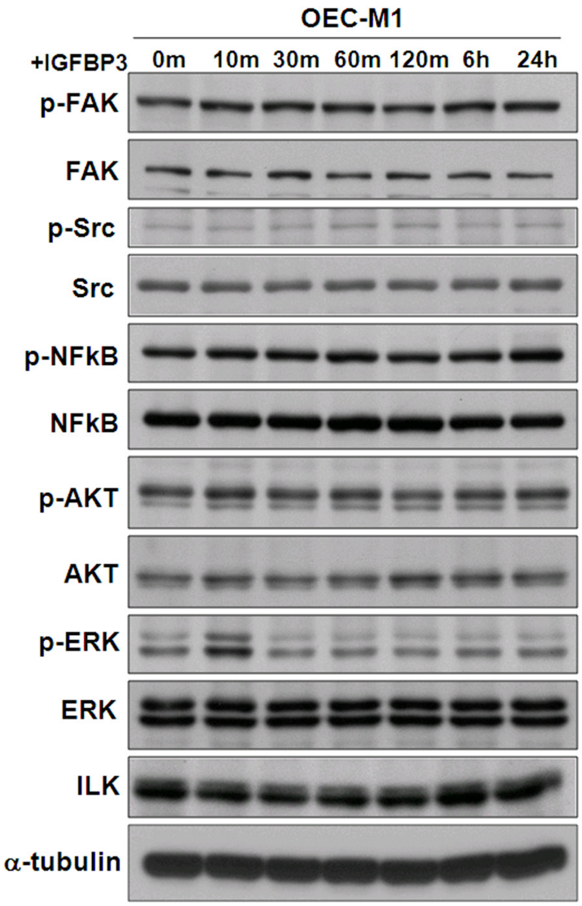

Supplementary Figure S6: Detection of ERK activation by IGFBP3 stimulation in OEC-M1 cells. Immunoblot assay demonstrated total and phosphorylated FAK/Src/NFkB/AKT/ERK and total ILK in OEC-M1 cells upon 100 ng/ml of IGFBP3 treatment at 10, 30, 60, 120 minutes, 6 and 24 hours. α-tubulin served as internal control for ILK expression.

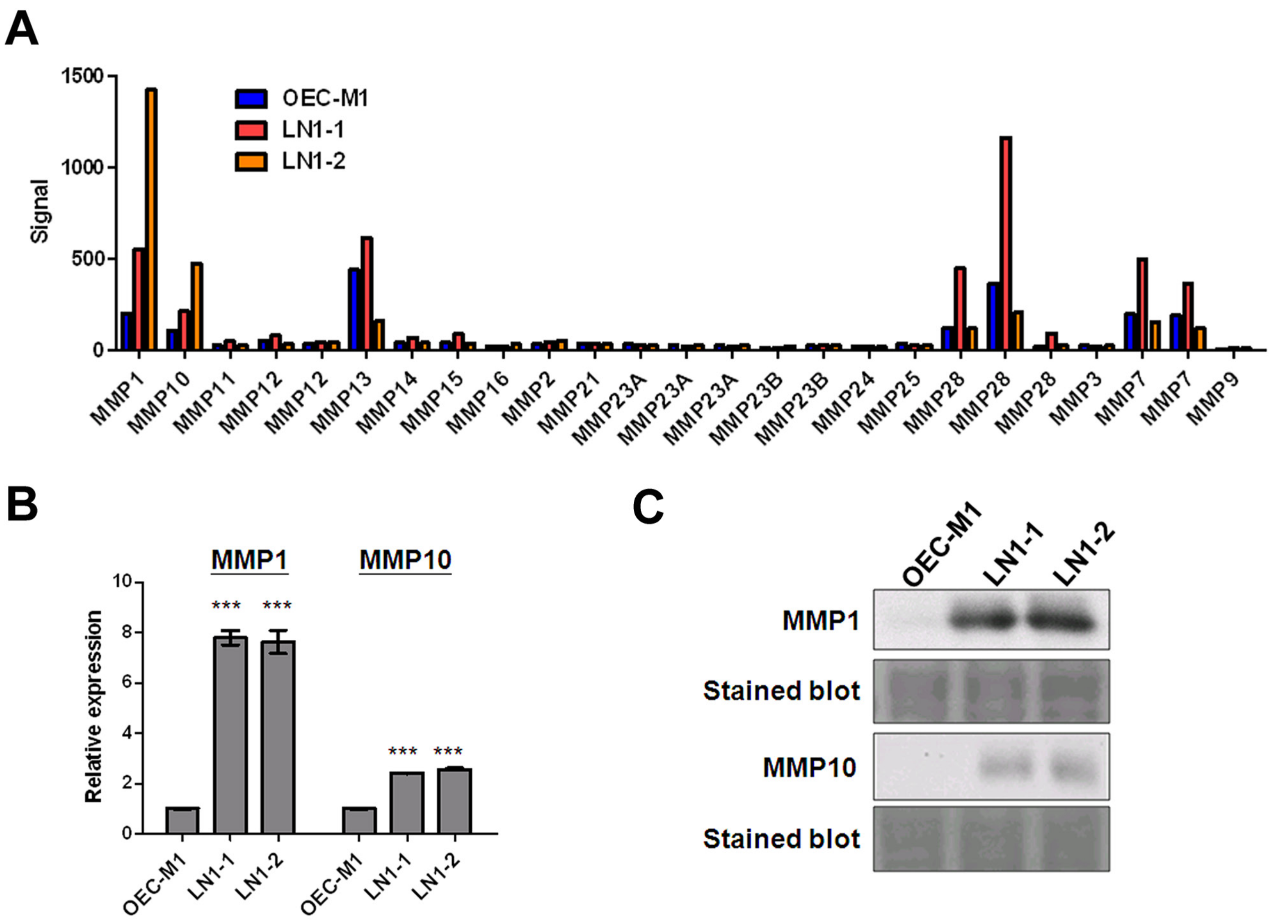

Supplementary Figure S7: Detection of MMP1 and MMP10 expression in OEC-M1, LN1–1 and LN1–2 cells. A. the signals of MMP-related gene expression were detected in OEC-M1, LN1–1 and LN1–2 cells by microarray analysis. **B.** Levels of MMP1 and MMP10 mRNA in OEC-M1, LN1–1 and LN1–2 cells were analyzed by qRT-PCR. All amplifications were normalized to an endogenous β-actin control. The relative expression of MMP mRNA in LN1–1 and -2 cells was normalized to that in OEC-M1 cells. **C.** Levels of MMP1 and MMP10 proteins in culture supernatant of OEC-M1, LN1–1 and LN1–2 cells were detected by Western blot. The stained blot served as protein loading controls. Bar, SE; ***$p<0.001$.

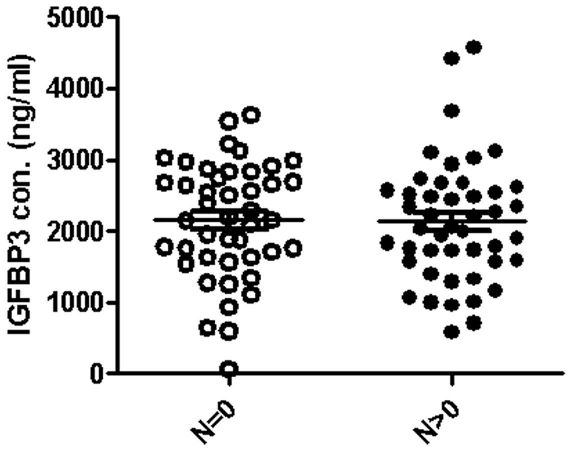

Supplementary Figure S8: Levels of IGFBP3 protein in plasma of OSCC patients were detected by ELISA. Levels of IGFBP3 protein did not show significant changes in plasma of OSCC patients with (n = 46) or without (n = 46) lymph node metastasis. Bar, SE
